# Dynamics of error-related activity in deterministic learning - an EEG and fMRI study

**DOI:** 10.1038/s41598-018-32995-x

**Published:** 2018-10-02

**Authors:** Magda Gawlowska, Aleksandra Domagalik, Ewa Beldzik, Tadeusz Marek, Justyna Mojsa-Kaja

**Affiliations:** 1 Institute of Applied Psychology, Jagiellonian University, Lojasiewicza 4, 30-348 Krakow, Poland; 2 Neuroimaging Group, Neurobiology Department, Malopolska Centre of Biotechnology, Jagiellonian University, Gronostajowa 7a, 30-347 Krakow, Poland

## Abstract

There is a close relationship between progress in learning and the error-monitoring system. EEG and fMRI studies using probabilistic learning have revealed the distinct dynamics of this system after response and feedback, i.e. an increase of error-related and a decrease of feedback-related activity in the anterior cingulate cortex (ACC). Based on the existing theories, it can be presumed that during deterministic learning feedback-related activity in ACC would also increase. Since these assumptions have not yet been confirmed, it can be only speculated based on the data from the probabilistic paradigms how the information is being integrated within the ACC during deterministic learning. Here we implemented the Paired Associate Deterministic Learning task to the EEG and fMRI experiments. The analysis of EEG data showed a significant increase in the amplitude for both ERN and FN. Similarly, the fMRI results showed an increase in response-related and feedback-related activity of the ACC in erroneous trials. Our findings are in line with the current theories of ACC function: increasing ACC activity can be linked to the detected discrepancy between expected and obtained outcomes. We argue that expectancy violations in the course of deterministic learning are signalled by both, internal and external evaluation system.

## Introduction

Learning is a process leading to relatively permanent change in behaviour as a result of practice or experience. Because an effective learning depends on the ability to adjust inefficient behaviour and build the most rewarding stimulus-outcome associations, it is inseparably related to monitoring for errors in actions. Action monitoring, and error detection in particular, are the processes guided by the anterior cingulate cortex (ACC)^1^ - region which receives dopaminergic signals from the midbrain, coding whether an event is better or worse than predicted^2^; therefore, it has been suggested that the ACC is involved in learning from errors^3^.

According to Shenhav and colleagues^4,5^ the ACC integrates a wide range of information about performance (including errors and feedback) in order to estimate the expected value of control (EVC), a quantity integrating the expected payoffs and costs of control signals. Alexander and Brown^6^ in their predicted response-outcome (PRO) model point that ACC may signal unexpected non-occurrences of predicted outcomes. Finally, the Action Value Updating model indicates that the outcome-related activity of ACC depends on the future usefulness of the outcome in guiding decisions, no matter the valence of feedback. Additionally, the response of ACC is stronger when the subject has already experienced negative feedback on preceding trials^7^.

The ACC has repeatedly been reported to be a source of generation of two event-related potentials (ERPs) observed in EEG recordings that are associated with error commission: error-related negativity (ERN) and feedback-related negativity (FN)^8–10^. ERN is a negative-going deflection which reaches its maximum amplitude approximately 60–100 ms after an incorrect response at the midline frontocentral scalp location^11,12^. ERN is considered a modality non-specific, generic part of the error-monitoring system which reflects internal evaluation of response correctness^13,14^. Its homolog component after correctly executed responses is correct response negativity (CRN), an event-related potential component topographically and morphologically similar to ERN, but of smaller amplitude^13^. Simultaneous recording of EEG and fMRI has shown that the magnitude of ERN correlates with the BOLD signal from the ACC on a trial-by-trial basis^8^. FN, the second of the potentials, is a negative-going ERP component which appears in EEG recordings 250–350 ms after external feedback about errors or losses is presented. Similar to ERN, FN has a midline frontocentral topography and, according to analysis of simultaneous EEG and fMRI data, is generated in the ACC^15^. FN is considered a reflection of an error signal when outcomes are valued as ‘worse than expected’^16–18^. When participants are presented with positive feedback, a positive-going ERP component called reward positivity (RewP) can be observed, with spatial and temporal properties similar to FN^19^.

The relationship between the learning process and the error-monitoring system has been extensively studied using both, the EEG and fMRI technique^20–24^ - however mostly in the probabilistic environment, as it is believed to better simulate the decision process and the probability of a favourable outcome in everyday life^25^. EEG studies show that probabilistic learning is associated with an inverse dynamic of change in ERN and FN amplitude over time, which reflects a shift between external and internal evaluation of the outcomes. Initially, the small ERN amplitude following a committed error increases as an individual learns about the associations between stimuli and correct reactions. On the other hand, the relatively high FN amplitude decreases with time due to the diminishing importance of the provided external evaluation of the response correctness^23^.

In the case of fMRI research, only a few studies focused on reporting the time-course of the activity over the learning process. Toni *et al*.^26^ used a visuomotor associative learning task and showed that the activity of ACC increases over the consecutive blocks of the task. Later, Law and colleagues^27^ reported brain regions that show either rising or dropping-off pattern of activity during the associative learning task. However, these studies report overlapping activities for response and for feedback and it is therefore not possible to conclude whether the changes in activity are related to changes in processing the correctness of response or with the assimilation of feedback information. Two separate studies examined the neural mechanisms involved in the error and feedback processing separately during learning in the probabilistic setting. Aron and colleagues^28^ compared brain activity on fixation, stimulus and feedback and showed greater ACC activity for the latter two. Mars *et al*.^29^ studied the dynamics of neural activity in probabilistic learning and found a pattern similar to the one from the EEG experiments, i.e. that feedback activity in the ACC was the greatest during the early phase of learning and gradually diminished later in the task. The opposite pattern was observed for the error-related activity of ACC.

In the neurocognitive research, deterministic learning has not drawn a lot of attention. As a matter of fact, studies implement deterministic learning paradigms mostly to compare it to the probabilistic environments, and not to study the rules of deterministic learning itself (e.g.^25,30–32^). Deterministic learning allows the application of a more analytic, rule-based mechanism of learning. It is easier to formulate a rule structure when the cue always correctly predicts the outcome^33^ - thus it substantially differs from the probabilistic one. To the best of our knowledge, there is no study directly exploring the dynamics of action-monitoring system activity in the relation to progress in learning in a fully predictable environment, and there is no empirical evidence whether the pattern of error-monitoring activity in the probabilistic learning holds true in the deterministic one.

Moreover, literature provides extensive data suggesting that the process of learning itself may be affected by the valence of feedback, but these results are not entirely consistent. According to Ashby and O’Brien^34^, people learn better when presented with negative feedback; however, there are also studies advocating the opposite, i.e. no difference in the effectiveness of learning from positive and negative feedback^35^, or even the opposite relation. For example, older people demonstrate the so-called ‘positive effect’: they learn better when presented with positive feedback^36^. Additionally, several other premises tend to treat the feedback of positive and negative value differently. As stated by Tversky & Kahneman^37^, in the prospect theory of loss aversion, loss is motivationally more significant than the equivalent gain. It has been shown that people prefer to receive feedback after a “good” rather than a “poor” trial^38^ as negative feedback is considered an irritating reminder that there is a need for a change of behaviour and improving the learning process^39^.

**Figure 1:**
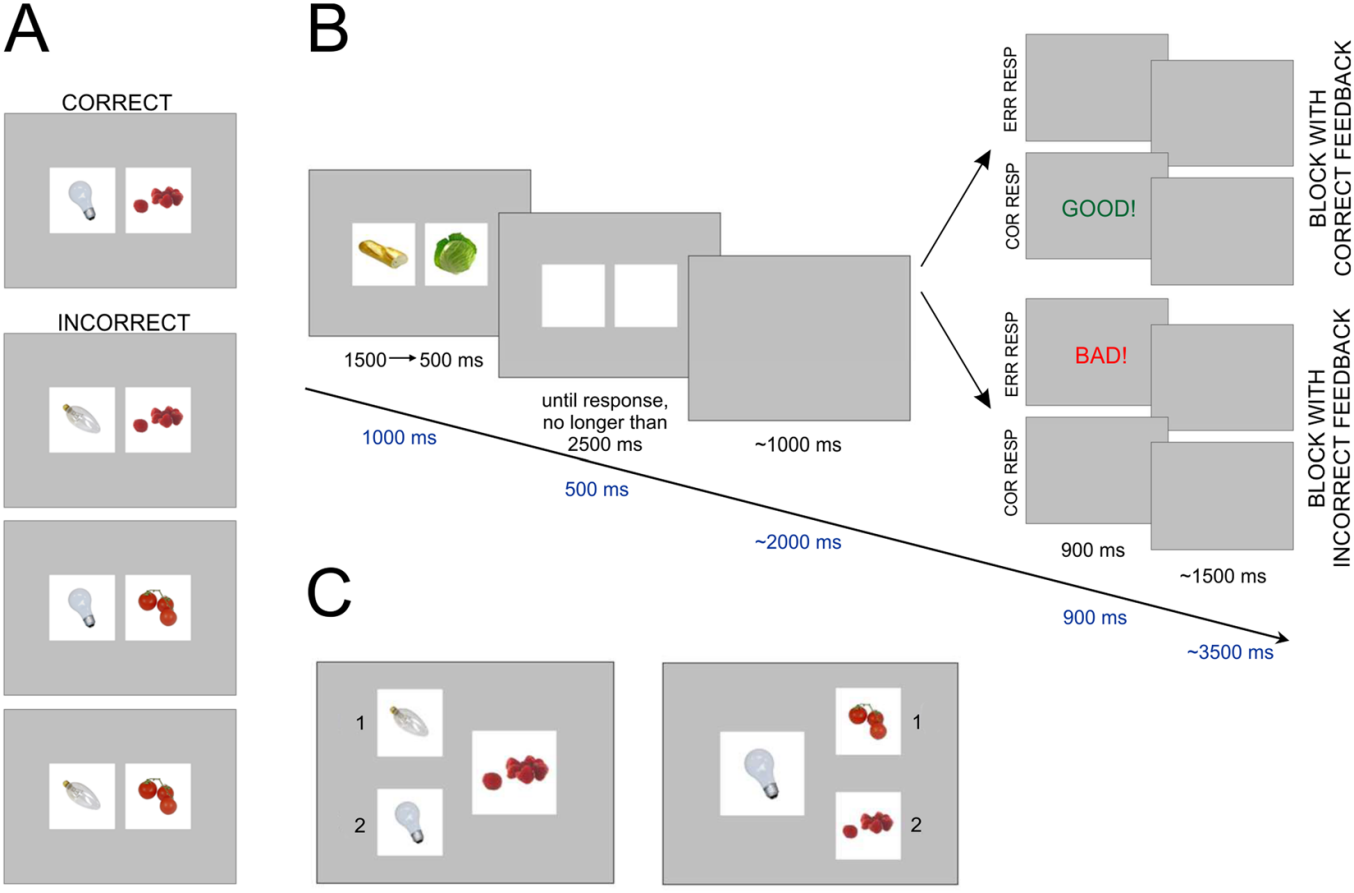
Experimental task design. (**A**) example of stimuli set; (**B**) experimental scheme (black numbers represent timing of presentation in the EEG experiment. Blue numbers represent timing of presentation in the fMRI experiment); (**C**) stimuli set from test phase. Image source: BOSS stimuli dataset^57^, distributed under the CC BY-SA license (https://creativecommons.org/licenses/by-sa/3.0/).

In the current study, we used the experimental paradigm we recently introduced which operates on the grounds of a deterministic environment:^40^ the Paired Associate Deterministic Learning task (PADL, Fig. 1). In our previous study, we showed that the PADL accurately models the relationship between progress in learning and multiple performance measures thus, we implement the PADL in EEG and fMRI experiments to evaluate the dynamics of error-monitoring system activity in terms of the ERN and FN components and the activity of the ACC over the process of deterministic learning. We manipulated timing parameters of the task procedure in order to separate activity related to response from activity related to feedback. To avoid the ‘double dipping’ problem^41^, in the analysis of EEG and fMRI data (i.e. the use of the same dataset firstly for selection and then for selective analysis) we predefined a set of EEG electrodes for extracting ERPs; brain regions of interest were derived from a meta-analysis database for BOLD signal measures. Based upon presented literature, we hypothesize that implementing the deterministic learning paradigm would reveal a specific pattern of learning process dynamics, i.e. both error and feedback-related activities will increase in the course of learning. If the obtained results are consistent with the hypothesis, this will provide strong support for existing ACC theories. Moreover, we introduce the positive and negative feedback conditions to explore their influence on the learning process in predictable environment.

## Materials and Methods

### Participants

Sixty-two young, neurotypical volunteers, recruited among the Jagiellonian University students through the website advertisement, participated in the study (32 females & 30 males; mean age 23.0 ± 2.3 years). Subjects met the experiment requirements: right-handedness, normal or corrected-to-normal vision, no neurological and psychiatric disorders and drug-free. Participants were financially rewarded for their participation, were informed about the procedure and goals of the study and gave their written informed consent. The study was approved by the Bioethics Commission at Jagiellonian University and all methods were performed in accordance with relevant guidelines and regulations.

From the initial group of sixty-two participants, EEG data analyses were performed on fifty-four subjects (29 females & 25 males; mean age 23 ± 2.4 years); four participants were excluded due to failure to comply with the task instruction and four participants were excluded due to extensive artifacts in the EEG recording.

Two participants dropped out after the EEG session. From the remaining sixty subjects, sixteen were excluded from fMRI data analysis due to missing data (i.e. no error trials in more than one learning time-point; 9 subjects; or incorrect button usage; 3 subjects), not achieving task goals (i.e. less than 80% of correct responses in the knowledge test; 3 subjects) or not finishing the task (1 subject). In the end, fMRI results were based on 44 subjects (20 females & 24 males; mean age 23.0 ± 2.2 years).

### Experimental procedure

The experiment was performed on three consecutive days: a practice session on the first day, an EEG recording session the next day and an MRI scanning session on the third day. During the practice run, participants were informed about the procedures, completed the consent form, read the task instructions and performed a full version of the PADL task with timing for the EEG experiment; they were informed that during fMRI procedure, the inter stimulus intervals would be longer.

During the EEG session (2nd day of the experiment) participants were presented with the task instruction again to ensure that they remembered the rules of the task. A 256-channel HydroCel Geodesic Sensor net was then set on each participant’s head. Stimuli were presented on a 19″ LCD monitor and subjects responded by pressing 1 or 2 on the Serial Response Box (©Psychology Software Tools) with the left and right index finger, respectively. Completing the entire EEG task took approximately 30 minutes.

In the fMRI session (3rd day of the experiment), subjects were presented with the task on a 32-inch screen located behind them, approximately 100 cm from a mirror placed on the head coil. Subjects responded by pressing a button on a Celeritas Fiber Optic Response System (©Psychology Software Tools) with the left or right index finger. The task in the fMRI session lasted about 1 h (details below).

### Experimental task

We used the recently introduced experimental paradigm^40^, which operates on the rules of a deterministic environment: the Paired Associate Deterministic Learning task (PADL). To account for the demands of the fMRI technique (task duration), we used only blocks with a monetary incentive. In the learning blocks, participants were presented with pairs of pictures and were asked to learn based on the presented feedback whether the pair was correct or not; they then submitted the answer by pressing 1 (“correct”) or 2 (“incorrect”) on the keypad. In every block, there were 9 unique stimuli sets. Each set comprised of 1 correct pair of pictures and 3 distracting pairs (Fig. 1A). The stimuli order was semi-randomized in order to distinguish 5 time-points of the learning process. Every time-point was marked by a single presentation of every correct pair and its variations (36 pairs in total). The two learning blocks differed in terms of feedback type. In the “positive” (POS) block, participants received feedback after correct responses and were informed that they would be able to win money in the test phase (up to 15 PLN, ~4$) if they matched pictures correctly. In the “negative” (NEG) block, participants received feedback only after incorrect responses. Moreover, they were informed they had been given 15 PLN, but they might lose it all if they matched pictures incorrectly in the test phase (Fig. 1B). Block order was counterbalanced across participants; half of the participants started with a NEG block and half with a POS block. Each test phase consisted of 18 boards (2 for each correct pair), and participants had to decide which picture (1 or 2) was part of the target stimulus (Fig. 1C).

Taking into consideration the low temporal resolution of fMRI, we optimized the timing parameter of the task procedure in order to be able to separate signals from response and feedback (see dark blue numbers below the arrow, Fig. 1B). The presentation of stimuli was 1000 ms for all learning time-points. Subjects had 1500 ms to give a response, after which feedback was shown for 900 ms. The time between stimulus offset and feedback presentation varied from 1000–3000 ms (average 2000 ms) and the time between feedback offset and the next stimuli was 2000–5000 ms (average 3500 ms). The EEG timing parameters were identical to the original version of the task (see black numbers above arrow, Fig. 1B). The sets of pictures were different for each session (i.e. practice, EEG and fMRI), but all sets contained equal number of stimuli.

Due to an insufficient number of committed errors, the fifth learning time-point had to be excluded from the EEG and the fMRI analysis. Thus, all the presented analyses of the EEG and fMRI data refer to 4 learning time-points, whereas the analysis of the behavioural data operates on 5 learning time-points.

### EEG data acquisition and analysis

Continuous dense-array EEG data (HydroCel Geodesic Sensor Net, EGI System 300; Electrical Geodesic Inc., OR, USA) was collected from 256 channel EEG at a sampling rate of 250 Hz (band-pass filtered at 0.01–100 Hz with a vertex electrode as a reference) and recorded with NetStation Software (Version 4.5.1, Electrical Geodesic Inc., OR, USA). The impedance for all electrodes was kept below 50 kΩ. Offline data analysis was conducted with the open-source EEGLAB toolbox (http://sccn.ucsd.edu/eeglab)^42^. Data was digitally filtered to remove frequencies below 0.5 Hz and above 35 Hz. Average reference was recomputed and bad channels were automatically removed by kurtosis measures with a threshold value of 5 standard deviations (on average 9.4% of the channels: FCz region was not affected). Continuous data was then visually inspected in order to manually remove remaining bad channels or time epochs containing high-amplitude, high-frequency muscle noise and other irregular artifacts.

Independent component analysis was used to remove artifacts from the data. Due to the large number of channels, decomposition of EEG data with the Infomax algorithm was preceded with Principal Component Analysis. Fifty independent components were extracted and visually inspected for each subject. On the basis of the spatiotemporal pattern^43,44^, components recognized as blinks, heart rate, saccades, muscle artifacts, or bad channels were removed. Missing channels were interpolated, and ICA weights were recomputed.

The response-related and feedback-related epochs were extracted from the data. The response-related epochs contained the time-window from 200 ms prior to the response, which served as a baseline and continued for 700 ms after the response. The feedback-related epochs contained the time-window from 200 ms prior to the feedback presentation, which served as a baseline and continued for 1000 ms following the feedback.

Following the previously applied methodology (e.g.)^45,46^, ERN amplitude was measured at the FCz electrode as a base-to-peak difference in voltage between the most negative peak from 0 to 150 ms after response onset and the preceding mean amplitude from −100 to 0 ms before response onset. To assess the amplitude of CRN, the same time-windows were applied to epochs containing correct responses.

FN amplitude measured at the FCz electrode was quantified as the difference in voltage between the most negative peak from 250 to 350 ms after negative feedback onset and the most positive peak from 150 to 250 ms after negative feedback onset. The same time-windows were applied to epochs containing positive feedback information (corresponding to RewP). This method of determining the amplitude of FN is not typical, especially when compared to the way FN is measured in gambling paradigms. The reason for the choice and application of the above method of FN quantification lies in the characteristics of the learning task used. As noted by Holroyd^47^, the measurements of FN amplitude tend to be distorted by the amplitude of the P300, a component FN is superimposed on. P300 amplitude is known to be strongly determined by the frequency of occurrence of the eliciting stimulus; therefore, the traditional base-to-peak measure of FN amplitude associated with frequent and infrequent stimuli would be contaminated by P300-related activity^16^.

### MRI data acquisition and analysis

Magnetic resonance imaging (MRI) was performed using a 3T scanner (Magnetom Skyra, Siemens) with a 20-channel head/neck coil. High-resolution, anatomical images were acquired using a T1 MPRAGE sequence (sagittal slices; 1 × 1 × 1.1 mm^3^ voxel size; TR = 2300 ms, TE = 2.98 ms).

Functional images were acquired using an EPI sequence. The scan parameters were as follows: 3 mm isotropic voxel, TR = 2000 ms, TE = 30 ms, flip angle = 90°, FOV 192 × 192 mm^2^, GRAPPA acceleration factor 2, phase encoding A/P. The whole brain image (cerebellum excluded) was covered with 35 axial slices, taken in an interleaved, ascending fashion. The acquisition time for each run was 24 minutes. There were two scanning runs, one for each task condition. The order of the conditions was counterbalanced across participants. Due to magnetic saturation effects, the first four volumes (dummy scans) of each run were acquired and then discarded by the scanner.

A standard pre-processing procedure was applied using Analysis of Functional NeuroImage (AFNI) software^48^. Each 3D image was time-shifted so the slices were aligned temporally. After head motion correction, the functional EPI data sets were coregistered to structural scans and smoothed using full-width at a half maximum isotropic Gaussian kernel of 4 mm. Voxels with low-signal intensity located outside the brain were excluded from the functional images by a clipping function. During the scaling procedure, the percent signal change was calculated for each voxel.

Higher level fMRI data analysis was carried out using FEAT (FMRI Expert Analysis Tool) Version 6.00, part of FSL (FMRIB’s Software Library, www.fmrib.ox.ac.uk/fsl). Time-series statistical analysis was carried out using FILM with local autocorrelation correction^49^. The occurrence of each specific trial, i.e. stimulus with subsequent correct response (COR), stimulus with subsequent erroneous response (ERR), stimulus without subsequent response, and feedback (FDB) for each learning block, was modelled using a box-car representation of the stimulus vector convolved with the canonical double-gamma model of the HRF and its temporal derivative. The duration of the box-car was equal to the duration of stimuli or feedback information occurrence. Additionally, 6 movement parameters were included in the model as nuisance regressors. After the subject-level statistical analysis, the normalization procedure was applied, i.e. anatomical images and beta maps were transformed into the coordinate system of MNI space. The parameter estimate corresponding to the level of activity was calculated using both the nonderivative and derivative term of the hemodynamic response function. This approach incorporates temporal derivatives to reduce the impact of spatially varying hemodynamic delays and delays caused by slice timing differences, as is more proper for testing amplitude differences^50^.

To avoid selection bias^41^, we defined the ACC as a region-of-interest using the Neurosynth meta-analytic database^51^ for both error and feedback analyses. The former was selected by searching for the term “error” (meta-analysis was automatically performed on 357 studies; centre-of-cluster MNI coordinates was 4, 27, 34). The latter was created using the custom meta-analysis option. The list of studies found after searching for the term “feedback” was verified and studies that did not present experiments on learning were excluded (e.g. we excluded studies on feedback in gambling tasks). In the end, 69 studies were included in “feedback-in-learning” meta-analysis (the list of studies is presented in Supplementary Table S1; centre-of-cluster MNI coordinates 3, 30, 38). Both reverse-inference maps were thresholded at q < 0.01 and FDR corrected. The ACC region was masked from both maps and then resampled to match the spatial resolution of the functional data. Parameter estimates for COR and ERR regressors in each block were extracted using the “error” map and the FDB regressor for each block was extracted from the “feedback-in-learning” map. Fourteen subjects did not have any errors in the third or fourth learning time-point of one of the task conditions; thus, for the missing data, the average values of the appropriate time-points were used as their parameters.

As an additional, confirmative step, we checked whether selected regions of interest overlapped with activity in the ACC in the current study. To do this, we created maps related to response and feedback regressors (averaged across all learning time-points and conditions). This analysis revealed full overlapping of ACC clusters (see Supplementary Fig. S2).

### Statistical analyses

To investigate the dynamics of EEG/fMRI activity on response, we used three-way repeated measures analysis of variance (ANOVA) with learning time-point (4 levels accounting for four learning time-points), condition (2 levels: POS vs. NEG) and accuracy (2 levels: COR vs. ERR) factors. The post-feedback EEG/fMRI activity was analysed with a two-way repeated measures ANOVA with the factors “learning time-point” (4 levels accounting for four learning time-points) and “condition” (2 levels: POS vs. NEG). Post hoc tests of simple effects were performed with the Tukey HSD test. All the statistical analyses were conducted with a significance threshold of 0.05.

All statistical testing was performed using Statistica 12 (Stat-Soft Inc) and Matlab 2015b (Mathworks, Inc.).

## Results

### Task performance

After each learning block, participants completed a test phase to ensure that the learning process had occurred. Data from test phase confirmed that participants had learned the pairs of objects. The percentage of correct responses was greater than 95% in both task sessions and both conditions (EEG, POS: 97.1% ± 0.6%, NEG: 97.5% ± 0.7%; fMRI, POS: 98.7% ± 0.5%, NEG: 99.5% ± 0.2%). These results suggest that participants gathered all the necessary knowledge in the learning process and, as a consequence, responded with close to perfect accuracy. The ANOVA test showed no difference in the correctness of the responses nether between the sessions (EEG vs. fMRI: F_(1, 96)_ = 3.85, *p* > 0.05), nor between the task conditions (negative vs. positive: F_(1, 96)_ = 1.46, *p* > 0.1). Moreover, there was no interaction effect of session and task condition (F_(1, 96)_ = 0.12, *p* > 0.7).

**Figure 2:**
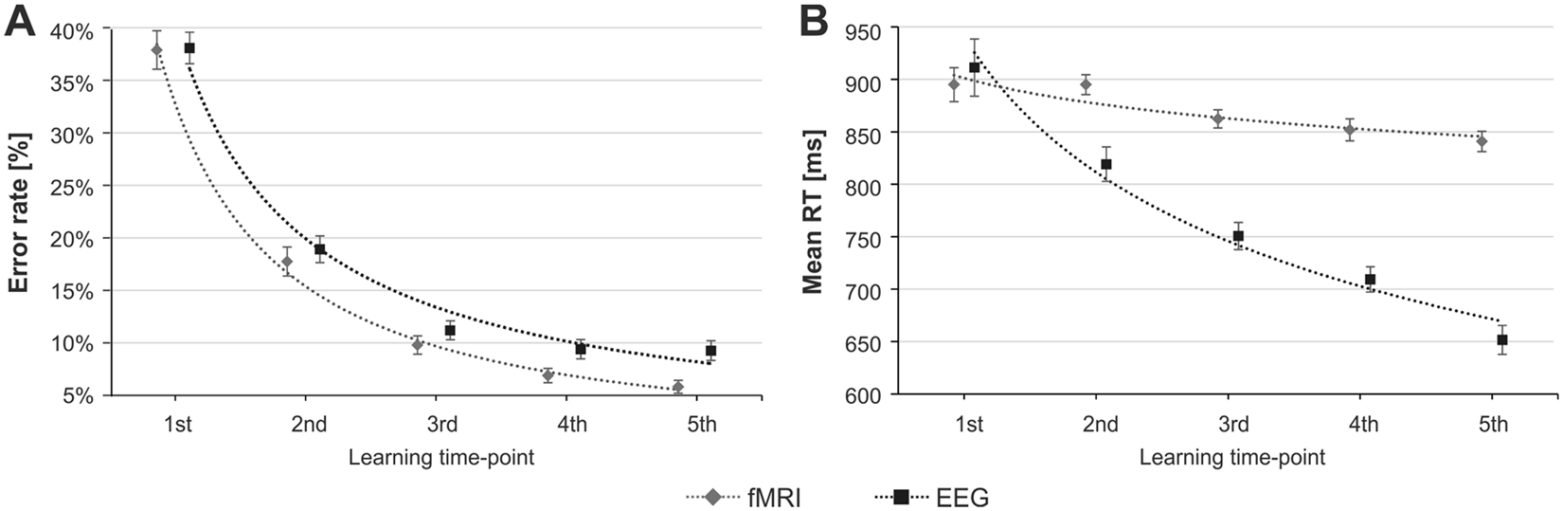
Task performance in EEG (black line) and fMRI (grey line) experiments: (**A**) mean error rate; (**B**) mean reaction time. Vertical bars denote standard error.

We observed a significant decrease in the error rates with the time of learning in both task sessions (EEG, F_(4, 212_) = 166.98, *p* < 0.001; fMRI, F_(4,172)_ = 167.58, *p* < 0.001; Fig. 2A). Moreover, there was no interaction effect of the task condition and session on error rate (F_(1, 96)_ = 0.00, *p* > 0.9). In both EEG and fMRI there was a decrease of the RT over the course of the learning (EEG, F_(4, 212)_ = 93.92, *p* < 0.001; fMRI, F_(4, 172)_ = 11.50, *p* < 0.001; Fig. 2B), and this decrease was not affected by the task condition – there was no significant interaction effect of the learning time-point and the task condition (F_(4, 384)_ = 0.70, *p* > 0.5).

To ensure the obtained results were not an effect of increased practice due to the repeated exposure to the experimental task, we compared EEG and fMRI error rates and RT. The error rates did not differ between the sessions (EEG vs. fMRI: F_(1, 96)_ = 2.70, *p* > 0.1). There was a significant difference in the RT between the session, with participants responding significantly slower while performing the task in the fMRI scanner (F_(1, 96)_ = 23.32, *p* < 0.001). This result is an effect of different timing parameters and specificity of used methods – i.e. longer intervals between the stimuli in the fMRI.

In both EEG and fMRI analysis, events were categorized as stimuli (targets and distractors) with subsequent correct (COR) or incorrect responses (ERR) and feedback (FDB); these were treated separately for task conditions and learning time-points. The fifth learning time-point was excluded from these analyses due to the insufficient number of incorrect responses.

### EEG results

**Figure 3:**
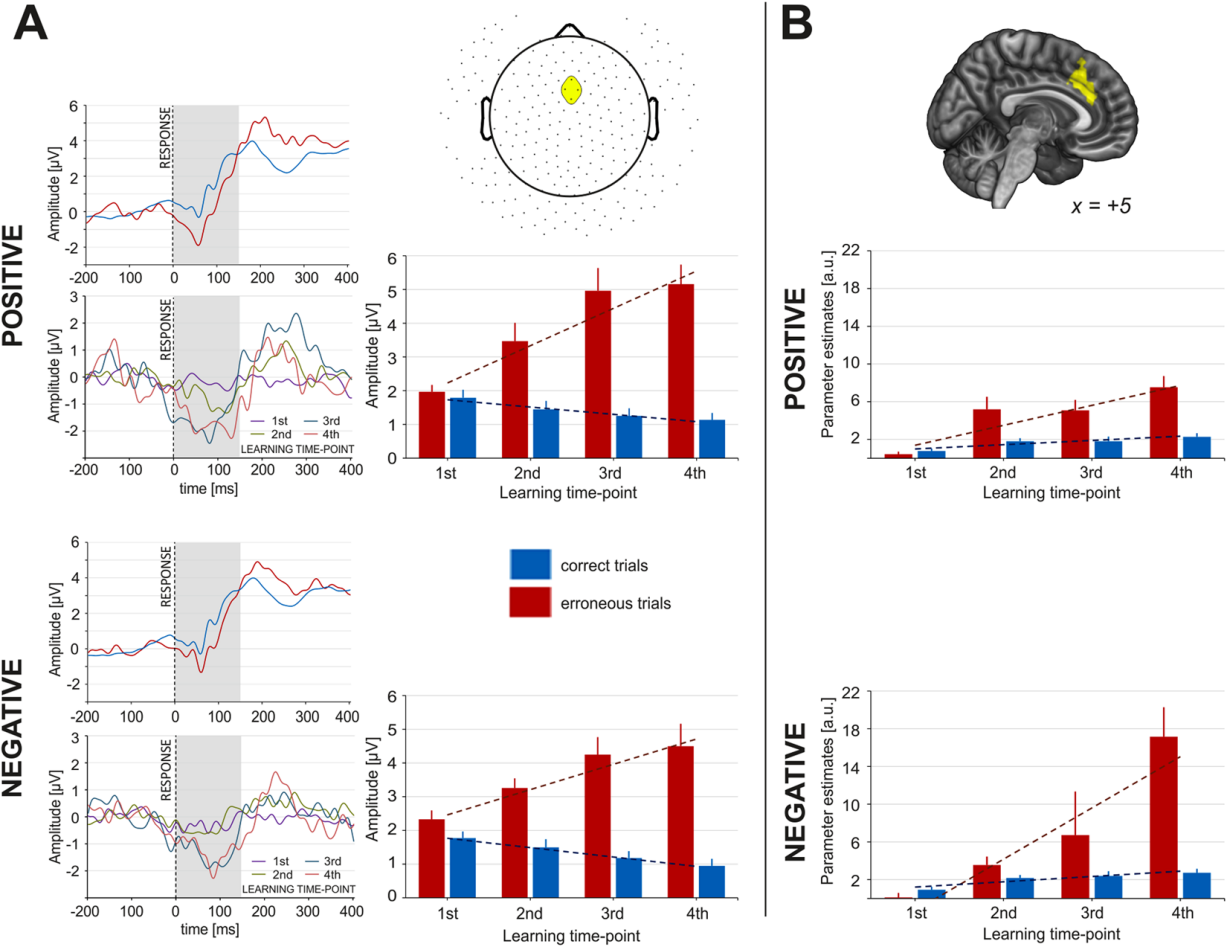
Response-related neural activity for blocks with positive and negative feedback. (**A**) results from EEG experiment: ERP plots averaged for 4 learning time-points (top left); delta ERN obtained by subtracting post-correct from post-error response activity for 4 learning time-points (bottom left); the absolute amplitude of ERN and CRN for 4 learning time-points (right); (**B**) results from fMRI experiment: response-related ACC activity for 4 learning time-points. Note: yellow colour represents the ROI for EEG and fMRI analysis (see Methods for details).

The response-related EEG activity was submitted to a three-way repeated measures ANOVA with accuracy (two levels: COR vs. ERR), condition (two levels: POS vs. NEG), and learning time-point (four levels) factors. We observed a main effect of response accuracy (F_(1, 53)_ = 111.96, *p* < 10^−6^, partial η^2^ = 0.68); EEG amplitude was significantly higher (i.e. more negative) after incorrect responses, which accounts for the ERN. Moreover, the analysis revealed a significant main effect of learning time-points (F_(3, 159)_ = 7.53, *p* < 0.001, partial η^2^ = 0.12), with post-response amplitude increasing with every subsequent learning time-point. Finally, there was an interaction effect of response accuracy and learning time-point (F_(3, 159)_ = 19.99, *p* < 10^−6^, partial η^2^ = 0.27). The post hoc analysis revealed a significant increase of ERN amplitude across the learning time-points, whereas the amplitude of CRN remained unaffected by the learning progress (see Fig. 3A).

**Figure 4:**
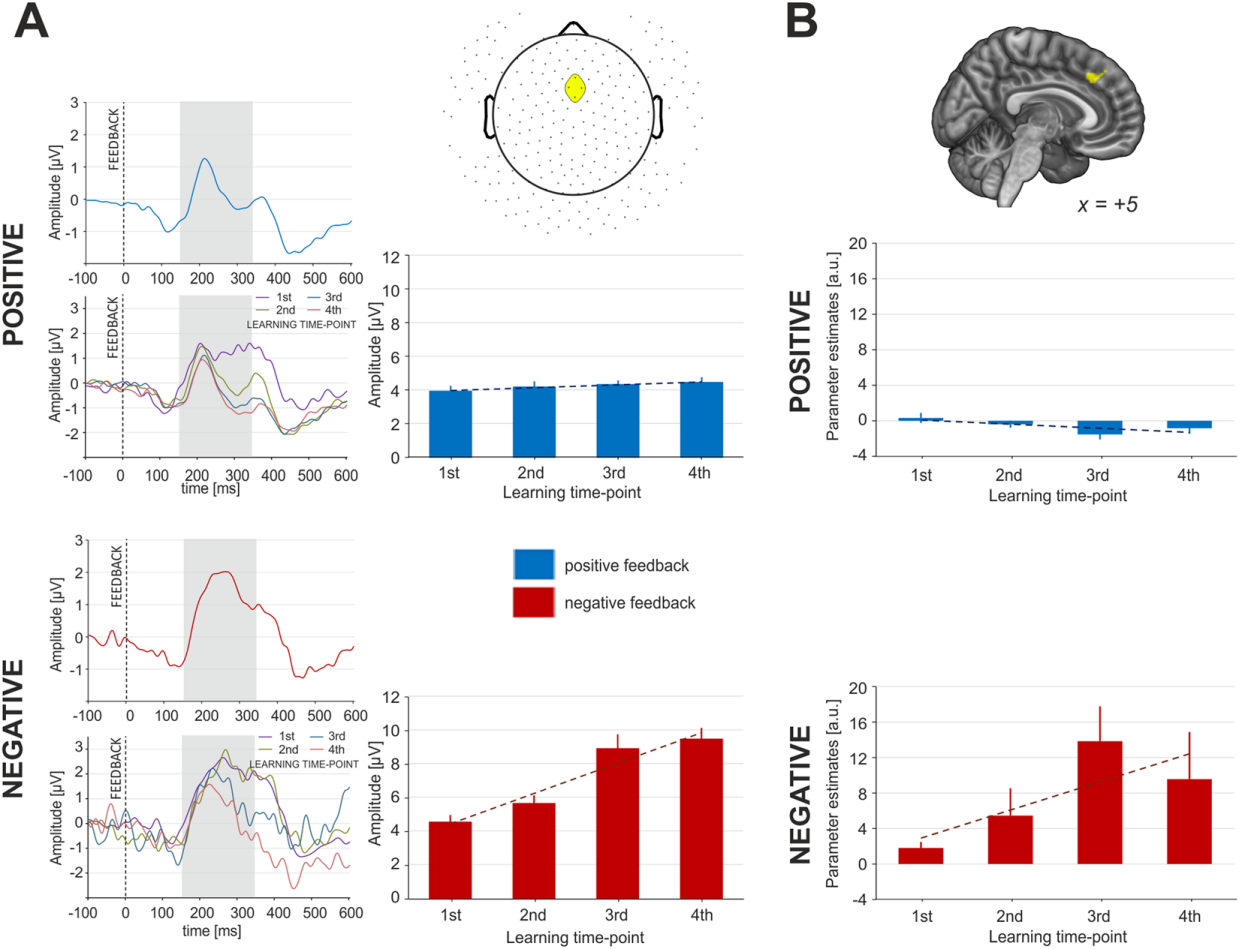
Feedback-related neural activity for blocks with positive and negative feedback. (**A**) results from EEG experiment: ERP plots averaged for 4 learning time-points (top left); ERP plots for 4 learning time-points separately (bottom left); the absolute amplitude of the post-feedback activity for 4 learning time-points (right); (**B**) results from fMRI experiment: feedback-related ACC activity for 4 learning time-points. Note: yellow colour represents the ROI for EEG and fMRI analysis (see Methods for details).

The feedback-related EEG activity was submitted to a two-way repeated measures ANOVA with condition (two levels: POS vs. NEG) and learning time-point (four levels) factors. We observed a main effect of condition (F_(1, 53)_ = 73.75, *p* < 10^−6^, partial η^2^ = 0.58) and learning time-point (F_(3, 159)_ = 22.37, *p* < 10^−6^, partial η^2^ = 0.30); the difference in the EEG amplitude was significantly higher after incorrect feedback, which accounts for FN and the increase in post-feedback EEG amplitude during learning progress. Moreover, the analysis revealed a significant interaction effect of condition and learning time-point (F_(3, 159)_ = 15.91, *p* < 10^−6^, partial η^2^ = 0.23). The post hoc analysis revealed a significant increase of FN amplitude across the learning time-points, whereas the amplitude of the RewP remained unaffected by the learning progress (see Fig. 4A).

Moreover, to test whether the presented results were not affected by the decreasing number of erroneous trials, we performed additional analysis on the randomly sampled data epochs (for details see the Supplementary Methods).

### fMRI results

Parameter estimates corresponding to the activation levels for COR, ERR and FDB in each learning time-point and condition were extracted from the ACC region defined using the Neurosynth meta-analytic database^51^. To investigate the dynamics of activity on response, we used a three-way repeated measures ANOVA with accuracy (two levels: COR vs. ERR), condition (two levels: POS vs. NEG) and learning time-point (four levels) factors. This analysis revealed significant higher activity of ACC for erroneous trials (Fig. 3B, F_(1, 43)_ = 25,69, *p* < 10^−6^, partial η^2^ = 0.37) and increasing ACC activity with the time of learning (Fig. 3B., F_(3, 129)_ = 13,96, *p* < 10^−6^, partial η^2^ = 0.25). Moreover, the analysis revealed a significant interaction between response accuracy and learning time-point (F_(3, 129)_ = 8,769, *p* < 0.001, partial η^2^ = 0.17). To investigate the dynamics of activity in response to feedback presentation, we used a two-way repeated measures ANOVA with condition (two levels: POS vs. NEG) and learning time-point (four levels) factors. The results showed that activity in response to feedback increased with the time of learning in the negative condition; for the positive condition, it remained stable and this activity was smaller in comparison to activity in response to negative feedback (Fig. 4B; F_(3,129)_ = 3.68, *p* < 0.02, partial η^2^ = 0.08).

## Discussion

The dynamics of ERN and FN and the activity of the ACC has been associated with learning progress in a probabilistic environment^29,52,53^. EEG research shows that over the course of the learning process, an inverse relationship between the amplitude of ERN and FN can be observed, which reflects a shift between external and internal evaluation of the outcomes^20–24^. At the beginning of the learning process, ERN, which is an indicator of the internal evaluation of erroneous responses, is characterized by a relatively small amplitude; as subjective knowledge increases, and the subject starts to rely on his/her own evaluation of events, an increase in the amplitude of ERN is also observed. In consequence, in the initial stage of learning there is a need to evaluate executed actions on the basis of external feedback. In these settings, external feedback presents great informative value which is reflected by an FN (an ERP component reflecting a neural response to negative, external feedback) of relatively high amplitude. As learning progresses, the amplitude of FN decreases due to the transition from external to internal evaluation of the action outcomes. Moreover, in probabilistic paradigms subject sees relatively quickly that feedback is not always a reliable source of information and that less attention should be paid to it. The dynamic characteristics of the interplay between external and internal sources of error information has also been shown in fMRI studies of probabilistic learning by emphasizing the contribution of the ACC to the selection of appropriate behaviours.

In our research, the learning process was carried out in a deterministic environment. The behavioural measures extracted from both the EEG and fMRI sessions, i.e. decreasing response time and error-rate, clearly show that participants were progressing in learning during the task. Taken together, as the number of correctly learned pairs assessed using the test phase of the task exceeded 95% in every condition and session, it may be concluded that the process of learning was successful and was the result of gathering all the necessary knowledge to perform well in the test phase.

Compared to tasks using probabilistic settings, analysis of the EEG and fMRI data from our task revealed different dynamics of the error-monitoring system. In both experiments, the results showed that instead of synchronously rising and falling in opposition to each other, the amplitudes of the ERN and FN components as well as the BOLD signal in the ACC for errors and negative feedback increased over the course of the learning. At the initial stage of learning, defined as the 1st learning time-point, the amplitude of ERN could not be distinguished from the amplitude of CRN (ΔERN, which reflects the error signal as it accounts for the difference between ERN and CRN, was close to zero μV); this was also true of responses related to ACC activity. This result suggests that participants had no knowledge about the correct and incorrect answers and they could not assess the outcomes of taken actions on the basis of internal evaluation. As learning progressed, the amplitude of ERN and the level of error-related ACC activity increased; at the same time, the amplitude of CRN and the level of ACC activity for correct responses remained stable.

Error signal carries the informative value, suggesting that the executed action was not suitable for the situational demands and there is a need to adjust behaviour. Correctly executed actions do not need adjustments, thus their informative value is substantially smaller and stable as learning progresses. The further into the learning process, the greater the activation in response to errors. The initial phase of learning is closely related to committing errors, as correct associations between stimuli, responses and outcomes have not been yet established. However, close to the end of the learning phase, when associations already exist, every error reflects a more severe and unexpected discrepancy between an expected and obtained outcome - thus becomes more ecologically salient information. This discrepancy generates a stronger error signal which informs there is a need to update the response strategy and implement immediate remedial actions. Thus, an increase in ERN and ACC activity for errors is a predictable result that has been presented in multiple previous studies^20–24^.

A deterministic environment significantly differs from a probabilistic one due to the nature of feedback. In probabilistic settings, feedback has to be processed cautiously due to its uncertain nature, whereas deterministic feedback always provides information coherent with the appropriateness of the executed action. When a learning process is more advanced, in probabilistic tasks participants already know that feedback does not always provide valuable information about the necessity to update the response strategy^30,54^; therefore, the error signal conveyed by the probabilistic feedback weakens with time. When deterministic feedback is considered, our data shows quite the opposite relationship. As the associations between stimuli, action and outcome are built on the basis of certain information, there is no space for error. The amplitude of FN increases for the same reason that the amplitude of ERN does: error reflects a more severe discrepancy between the expected and obtained outcome and, as a consequence, a stronger error signal. The same situation is observed when looking at ACC activity. Both error-related and negative feedback-related activity increase in consecutive learning time-points. As suggested by Arbel and colleagues^18^, the magnitude of the response to presented feedback may represent not only its value (i.e. “better or worse than expected”), but also its utility in the process of learning; committing an error in the final phase of the learning process indicates still present deficits in stimulus-response mappings. The study by Aarts and colleagues^55^ supports this explanation: they showed that activity of ACC, as involved in anticipatory control, is larger after informative than uninformative cues.

Moreover, the amplitude of RewP and ACC activity for positive feedback was not sensitive to the progress of the learning process. This fact may be explained on the same grounds as the constant amplitude of CRN which we observed throughout the learning process. As positive feedback informs about a correctly executed action, there is no need to adjust the behaviour and the informative value of positive feedback remains stable throughout the process of learning; it simply informs, “continue doing what you are doing”.

The above results support the existing theories of the ACC function. For example, considering the PRO model^6^, increased ACC activity reflected in rising amplitude of the ERN and FN and BOLD signal for errors and negative feedback, may indicate the absence of positive, expected outcomes. As the learning at its final stages is advanced, one expects that submitted responses would be evaluated as correct. In consequence, errors may be considered not as a negative outcome, but as a lack of positive, expected one. Further, positive outcomes (i.e. correct responses and positive feedback information), are expected due to the obtained mastery in the task, thus they are not likely to result in an increase of the ACC activity. Similarly, the Action Value Updating model^7^ points at the usefulness of feedback in the future. At the final stages of learning correct responses are no longer a useful source of information, unlike the erroneous ones.

Interestingly, the introduced positive and negative conditions did not influence the performance. Also, there was no significant difference in post-response data. On the contrary, the post-feedback EEG data and the ACC activity for feedback were sensitive to feedback valence modulation. The ACC activity was higher in the negative block, suggesting greater ACC involvement when there is a need to adjust the response strategy, compared to situations in which a certain pattern of behaviour should be continued^10,56^. Similarly, the post-feedback EEG activity shows a greater increase in FN amplitude when participants are presented with negative feedback information.

Finally, one may notice that as learning progressed, the number of errors dramatically deceased to reach less than 10% in the 4th learning time-point. Aware of the possible distorting effect of the unequal number of trials and its potential influence on presented EEG results, we performed additional analysis with a balanced number of errors across the learning time-points (see the Supplementary Methods for details). The obtained results show that even when the number of errors is reduced to approximately 10% in every learning time-point, the effects we describe hold - thus, the noise-driven, false-positive results may be ruled out.

To sum up, we studied the neural dynamics of an error monitoring system during learning in a deterministic environment using both EEG and fMRI techniques. We were able to separate the response- and feedback-related BOLD signals and explored changes in its amplitude as well as in the amplitude of response and feedback related EEG potentials. The complementary and supportive findings of the two experiments reveal that activity for incorrect responses and negative feedback increases over the course of learning. Considering the differences between probabilistic and deterministic learning processes, the obtained results suggest similar engagement of internal and external evaluation systems in uncovering and reacting to errors committed during the latter. Despite the different nature of the deterministic learning process, these results provide support for the current theories of error-processing within ACC.

## Electronic supplementary material

Supplementary Information

## Data Availability

The datasets collected and analysed during the current study are available upon request from the corresponding author.
