# Supplementary material for: Dynamics of error-related activity in deterministic learning - an EEG and fMRI study
Source: Sci Rep. 2018 Oct 2;8:14617. doi: 10.1038/s41598-018-32995-x (PMC6168565; doi:10.1038/s41598-018-32995-x)
Supplement: Supplementary file 1 — Supplementary Information [file 41598_2018_32995_MOESM1_ESM.pdf]

# Dynamics of error-related activity in deterministic learning - an EEG and fMRI study.

Magda Gawlowska[1,*,+], Aleksandra Domagalik[2,+], Ewa Beldzik[1], Tadeusz Marek[1], and Justyna Mojsa-Kaja[1]

[1] Institute of Applied Psychology, Jagiellonian University, Lojasiewicza 4, 30-348 Krakow, Poland

[2] Neuroimaging Group, Neurobiology Department, Malopolska Centre of Biotechnology, Jagiellonian University, Gronostajowa 7a, 30-347 Krakow, Poland

[+] these authors contributed equally to this work

*** Corresponding Author:**

Magda Gawlowska,

Institute of Applied Psychology, Jagiellonian University, Lojasiewicza 4, 30-348 Krakow, Poland

e-mail: magda.gawlowska@uj.edu.pl

**Supplementary Table S1** Studies from the Neurosynth meta-analytic database used in the "feedback-in-learning" meta-analysis to define the ACC as a region-of-interest.

| | AUTHOR | TITLE | JOURNAL |
|---|---|---|---|
| 1 | Amiez C, Neveu R, Warrot D, Petrides M, Knoblauch K, Procyk E | The location of feedback-related activity in the midcingulate cortex is predicted by local morphology. | *Journal of Neuroscience*, *33*(5), 2217-2228. |
| 2 | Amiez C, Sallet J, Procyk E, Petrides M | Modulation of feedback related activity in the rostral anterior cingulate cortex during trial and error exploration. | *Neuroimage*, *63*(3), 1078-1090. |
| 3 | Appelgren A, Bengtsson SL | Feedback on Trait or Action Impacts on Caudate and Paracingulum Activity. | *PloS one*, *10*(6), e0129714. |
| 4 | Aron AR, Shohamy D, Clark J, Myers C, Gluck MA, Poldrack RA | Human midbrain sensitivity to cognitive feedback and uncertainty during classification learning. | *Journal of neurophysiology*, *92*(2), 1144-1152. |
| 5 | Becker MP, Nitsch AM, Schlosser R, Koch K, Schachtzabel C, Wagner G, Miltner WH, Straube T | Altered emotional and BOLD responses to negative, positive and ambiguous performance feedback in OCD. | *Social cognitive and affective neuroscience*, *9*(8), 1127-1133. |
| 6 | Bellebaum C, Jokisch D, Gizewski ER, Forsting M, Daum I | The neural coding of expected and unexpected monetary performance outcomes: dissociations between active and observational learning. | *Behavioural brain research*, *227*(1), 241-251. |
| 7 | Bischoff-Grethe A, Hazeltine E, Bergren L, Ivry RB, Grafton ST | The influence of feedback valence in associative learning. | *Neuroimage*, *44*(1), 243-251 |
| 8 | Boorman ED, Rushworth MF, Behrens TE | Ventromedial prefrontal and anterior cingulate cortex adopt choice and default reference frames during sequential multi-alternative choice. | *Journal of Neuroscience*, *33*(6), 2242-2253. |
| 9 | Carlson JM, Foti D, Mujica-Parodi LR, Harmon-Jones E, Hajcak G | Ventral striatal and medial prefrontal BOLD activation is correlated with reward-related electrocortical activity: a combined ERP and fMRI study. | *Neuroimage*, *57*(4), 1608-1616. |
| 10 | Chaumon M, Kveraga K, Barrett LF, Bar M | Visual Predictions in the Orbitofrontal Cortex Rely on Associative Content. | *Cerebral Cortex*, *24*(11), 2899-2907. |
| 11 | Chua HF, Gonzalez R, Taylor SF, Welsh RC, Liberzon I | Decision-related loss: regret and disappointment. | *Neuroimage*, *47*(4), 2031-2040. |
| 12 | Cohen MX, Elger CE, Weber B | Amygdala tractography predicts functional connectivity and learning during feedback-guided decision-making. | *Neuroimage*, *39*(3), 1396-1407. |
| 13 | Cools R, Clark L, Owen AM, Robbins TW | Defining the neural mechanisms of probabilistic reversal learning using event-related functional magnetic resonance imaging. | *Journal of Neuroscience*, *22*(11), 4563-4567. |
| 14 | Daniel R, Pollmann S | Comparing the neural basis of monetary reward and cognitive feedback during information-integration category learning. | *Journal of Neuroscience*, *30*(1), 47-55. |
| 15 | Daniel R, Pollmann S | Striatal activations signal prediction errors on confidence in the absence of external feedback. | *Neuroimage*, *59*(4), 3457-3467. |
| 16 | D'Cruz AM, Ragozzino ME, Mosconi MW, Pavuluri MN, Sweeney JA | Human reversal learning under conditions of certain versus uncertain outcomes. | *Neuroimage*, *56*(1), 315-322. |

| | | | |
|---|---|---|---|
| 17 | Debaere F, Wenderoth N, Sunaert S, Van Hecke P, Swinnen SP | Internal vs external generation of movements: differential neural pathways involved in bimanual coordination performed in the presence or absence of augmented visual feedback. | *Neuroimage*, *19*(3), 764-776. |
| 18 | Delgado MR, Nystrom LE, Fissell C, Noll DC, Fiez JA | Tracking the hemodynamic responses to reward and punishment in the striatum. | *Journal of neurophysiology*, *84*(6), 3072-3077. |
| 19 | Dibbets P, Evers L, Hurks P, Marchetta N, Jolles J | Differences in feedback- and inhibition-related neural activity in adult ADHD. | *Brain and Cognition*, *70*(1), 73-83. |
| 20 | Eliassen JC, Lamy M, Allendorfer JB, Boespflug E, Bullard DP, Smith MS, Lee JH, Strakowski SM | Selective role for striatal and prefrontal regions in processing first trial feedback during single-trial associative learning. | *Brain research*, *1458*, 56-66. |
| 21 | Goldhacker M, Rosengarth K, Plank T, Greenlee MW | The effect of feedback on performance and brain activation during perceptual learning. | *Vision research*, *99*, 99-110. |
| 22 | Greening SG, Finger EC, Mitchell DG | Parsing decision making processes in prefrontal cortex: response inhibition, overcoming learned avoidance, and reversal learning. | *Neuroimage*, *54*(2), 1432-1441. |
| 23 | Hamann JM, Dayan E, Hummel FC, Cohen LG | Baseline frontostriatal-limbic connectivity predicts reward-based memory formation. | *Human brain mapping*, *35*(12), 5921-5931. |
| 24 | Hauser TU, Iannaccone R, Stampfli P, Drechsler R, Brandeis D, Walitza S, Brem S | The feedback-related negativity (FRN) revisited: new insights into the localization, meaning and network organization. | *Neuroimage*, *84*, 159-168. |
| 25 | Hauser TU, Iannaccone R, Walitza S, Brandeis D, Brem S | Cognitive flexibility in adolescence: Neural and behavioral mechanisms of reward prediction error processing in adaptive decision making during development. | *Neuroimage*, *104*, 347-354. |
| 26 | Helfinstein SM, Benson B, Perez-Edgar K, Bar-Haim Y, Detloff A, Pine DS, Fox NA, Ernst M | Striatal responses to negative monetary outcomes differ between temperamentally inhibited and non-inhibited adolescents. | *Neuropsychologia*, *49*(3), 479-485. |
| 27 | Hester R, Barre N, Murphy K, Silk TJ, Mattingley JB | Human medial frontal cortex activity predicts learning from errors. | *Cerebral Cortex*, *18*(8), 1933-1940. |
| 28 | Javadi AH, Schmidt DH, Smolka MN | Differential representation of feedback and decision in adolescents and adults. | *Neuropsychologia*, *56*, 280-288. |
| 29 | Jiang Y, Kim SI, Bong M | Effects of reward contingencies on brain activation during feedback processing. | *Frontiers in human neuroscience*, *8*. |
| 30 | Jimura K, Konishi S, Miyashita Y | Dissociable concurrent activity of lateral and medial frontal lobe during negative feedback processing. | *Neuroimage*, *22*(4), 1578-1586. |
| 31 | Jones RM, Somerville LH, Li J, Ruberry EJ, Powers A, Mehta N, Dyke J, Casey BJ | Adolescent-specific patterns of behavior and neural activity during social reinforcement learning. | *Cognitive, Affective, & Behavioral Neuroscience*, *14*(2), 683-697. |
| 32 | Joseph JE, Zhu X, Corbly CR, DeSantis S, Lee DC, Baik G, Kiser S, Jiang Y, Lynam DR, Kelly TH | Influence of neurobehavioral incentive valence and magnitude on alcohol drinking behavior. | *NeuroImage*, *104*, 373-385. |
| 33 | Kirsch P, Schienle A, Stark R, Sammer G, Blecker C, Walter B, Ott U, Burkart J, Vaitl D | Anticipation of reward in a nonaversive differential conditioning paradigm and the brain reward system: an event-related fMRI study. | *Neuroimage*, *20*(2), 1086-1095. |

| | | | |
|---|---|---|---|
| 34 | Koch K, Schachtzabel C, Wagner G, Schikora J, Schultz C, Reichenbach JR, Sauer H, Schlosser RG | Altered activation in association with reward-related trial-and-error learning in patients with schizophrenia. | *Neuroimage*, *50*(1), 223-232. |
| 35 | Koolschijn PC, Schel MA, de Rooij M, Rombouts SA, Crone EA | A three-year longitudinal functional magnetic resonance imaging study of performance monitoring and test-retest reliability from childhood to early adulthood. | *Journal of Neuroscience*, *31*(11), 4204-4212 |
| 36 | Korn CW, Fan Y, Zhang K, Wang C, Han S, Heekeren HR | Cultural influences on social feedback processing of character traits. | *Frontiers in human neuroscience*, *8*. |
| 37 | Kotani Y, Ohgami Y, Kuramoto Y, Tsukamoto T, Inoue Y, Aihara Y | The role of the right anterior insular cortex in the right hemisphere preponderance of stimulus-preceding negativity (SPN): an fMRI study. | *Neuroscience letters*, *450*(2), 75-79. |
| 38 | Labudda K, Woermann FG, Mertens M, Pohlmann-Eden B, Markowitsch HJ, Brand M | Neural correlates of decision making with explicit information about probabilities and incentives in elderly healthy subjects. | *Experimental Brain Research*, *187*(4), 641-650. |
| 39 | Lam JM, Wachter T, Globas C, Karnath HO, Luft AR | Predictive value and reward in implicit classification learning. | *Human brain mapping*, *34*(1), 176-185. |
| 40 | Lee W, Kim SI | Effects of achievement goals on challenge seeking and feedback processing: behavioral and FMRI evidence. | *PloS one*, *9*(9), e107254. |
| 41 | Linke J, Kirsch P, King AV, Gass A, Hennerici MG, Bongers A, Wessa M | Motivational orientation modulates the neural response to reward. | *Neuroimage*, *49*(3), 2618-2625. |
| 42 | Little DM, Shin SS, Sisco SM, Thulborn KR | Event-related fMRI of category learning: differences in classification and feedback networks. | *Brain and cognition*, *60*(3), 244-252. |
| 43 | Little DM, Thulborn KR | Correlations of cortical activation and behavior during the application of newly learned categories. | *Cognitive Brain Research*, *25*(1), 33-47. |
| 44 | Mars RB, Coles MG, Grol MJ, Holroyd CB, Nieuwenhuis S, Hulstijn W, Toni I | Neural dynamics of error processing in medial frontal cortex. | *Neuroimage*, *28*(4), 1007-1013. |
| 45 | Muller SV, Moller J, Rodriguez-Fornells A, Munte TF | Brain potentials related to self-generated and external information used for performance monitoring. | *Clinical Neurophysiology*, *116*(1), 63-74. |
| 46 | Nees F, Vollstadt-Klein S, Fauth-Buhler M, et al. | A target sample of adolescents and reward processing: same neural and behavioral correlates engaged in common paradigms? | *Experimental brain research*, *223*(3), 429-439. |
| 47 | Nieuwenhuis S, Slagter HA, von Geusau NJ, Heslenfeld DJ, Holroyd CB | Knowing good from bad: differential activation of human cortical areas by positive and negative outcomes. | *European Journal of Neuroscience*, *21*(11), 3161-3168. |
| 48 | O'Connor DA, Rossiter S, Yucel M, Lubman DI, Hester R | Successful inhibitory control over an immediate reward is associated with attentional disengagement in visual processing areas. | *Neuroimage*, *62*(3), 1841-1847. |
| 49 | Papo D, Douiri A, Bouchet F, Bourzeix JC, Caverni JP, Baudonniere PM | Time-frequency intracranial source localization of feedback-related EEG activity in hypothesis testing. | *Cerebral Cortex*, *17*(6), 1314-1322. |
| 50 | Remijnse PL, Nielen MM, Uylings HB, Veltman DJ | Neural correlates of a reversal learning task with an affectively neutral baseline: an event-related fMRI study. | *Neuroimage*, *26*(2), 609-618 |

| | | | |
|---|---|---|---|
| 51 | Sailer U, Robinson S, Fischmeister FP, Konig D, Oppenauer C, Lueger-Schuster B, Moser E, Kryspin-Exner I, Bauer H | Altered reward processing in the nucleus accumbens and mesial prefrontal cortex of patients with posttraumatic stress disorder. | *Neuropsychologia*, *46*(11), 2836-2844. |
| 52 | Sailer U, Robinson S, Fischmeister FP, Moser E, Kryspin-Exner I, Bauer H | Imaging the changing role of feedback during learning in decision-making. | *Neuroimage*, *37*(4), 1474-1486. |
| 53 | Santesso DL, Dillon DG, Birk JL, Holmes AJ, Goetz E, Bogdan R, Pizzagalli DA | Individual differences in reinforcement learning: behavioral, electrophysiological, and neuroimaging correlates. | *Neuroimage*, *42*(2), 807-816. |
| 54 | Schott BH, Niehaus L, Wittmann BC, Schutze H, Seidenbecher CI, Heinze HJ, Duzel E | Ageing and early-stage Parkinson's disease affect separable neural mechanisms of mesolimbic reward processing. | *Brain*, *130*(9), 2412-2424. |
| 55 | Seger CA, Cincotta CM | The roles of the caudate nucleus in human classification learning. | *Journal of Neuroscience*, *25*(11), 2941-2951. |
| 56 | Spielberg JM, Jarcho JM, Dahl RE, Pine DS, Ernst M, Nelson EE | Anticipation of peer evaluation in anxious adolescents: divergence in neural activation and maturation. | *Social cognitive and affective neuroscience*, *10*(8), 1084-1091. |
| 57 | Spreckelmeyer KN, Krach S, Kohls G, Rademacher L, Irmak A, Konrad K, Kircher T, Grunder G | Anticipation of monetary and social reward differently activates mesolimbic brain structures in men and women. | *Social cognitive and affective neuroscience*, *4*(2), 158-165. |
| 58 | Stark R, Bauer E, Merz CJ, Zimmermann M, Reuter M, Plichta MM, et al. | ADHD related behaviors are associated with brain activation in the reward system. | *Neuropsychologia*, *49*(3), 426-434. |
| 59 | Tavares JVT, Clark L, Furey ML, Williams GB, Sahakian BJ, Drevets WC | Neural basis of abnormal response to negative feedback in unmedicated mood disorders. | *Neuroimage*, *42*(3), 1118-1126. |
| 60 | Tricomi E, Fiez JA | Information content and reward processing in the human striatum during performance of a declarative memory task. | *Cognitive, Affective, & Behavioral Neuroscience*, *12*(2), 361-372. |
| 61 | Tsuchida A, Doll BB, Fellows LK | Beyond reversal: a critical role for human orbitofrontal cortex in flexible learning from probabilistic feedback. | *Journal of Neuroscience*, *30*(50), 16868-16875. |
| 62 | van den Bos W, Cohen MX, Kahnt T, Crone EA | Striatum-medial prefrontal cortex connectivity predicts developmental changes in reinforcement learning. | *Cerebral Cortex*, *22*(6), 1247-1255. |
| 63 | Vickery TJ, Jiang YV | Inferior parietal lobule supports decision making under uncertainty in humans. | *Cerebral Cortex*, *19*(4), 916-925. |
| 64 | Volz KG, Schubotz RI, von Cramon DY | Frontomedian activation depends on both feedback validity and valence: fMRI evidence for contextual feedback evaluation. | *NeuroImage*, *27*(3), 564-571. |
| 65 | Waldschmidt JG, Ashby FG | Cortical and striatal contributions to automaticity in information-integration categorization. | *Neuroimage*, *56*(3), 1791-1802. |
| 66 | Walsh ND, Phillips ML | Interacting outcome retrieval, anticipation, and feedback processes in the human brain. | *Cerebral Cortex*, *20*(2), 271-281. |

| | | | |
|---|---|---|---|
| 67 | Yomogida Y, Sugiura M, Sassa Y, et al. | The neural basis of agency: an fMRI study. | *Neuroimage*, *50*(1), 198-207. |
| 68 | Zanolie K, Teng S, Donohue SE, van Duijvenvoorde AC, Band GP, et al. | Switching between colors and shapes on the basis of positive and negative feedback: an fMRI and EEG study on feedback-based learning. | *Cortex*, *44*(5), 537-547. |
| 69 | Zanolie K, Van Leijenhorst L, Rombouts SA, Crone EA | Separable neural mechanisms contribute to feedback processing in a rule-learning task. | *Neuropsychologia*, *46*(1), 117-126. |

**Supplementary Figure S2** Results of GLM analysis (red) of (A) response and (B) feedback with overlapping selected regions of interest (yellow).

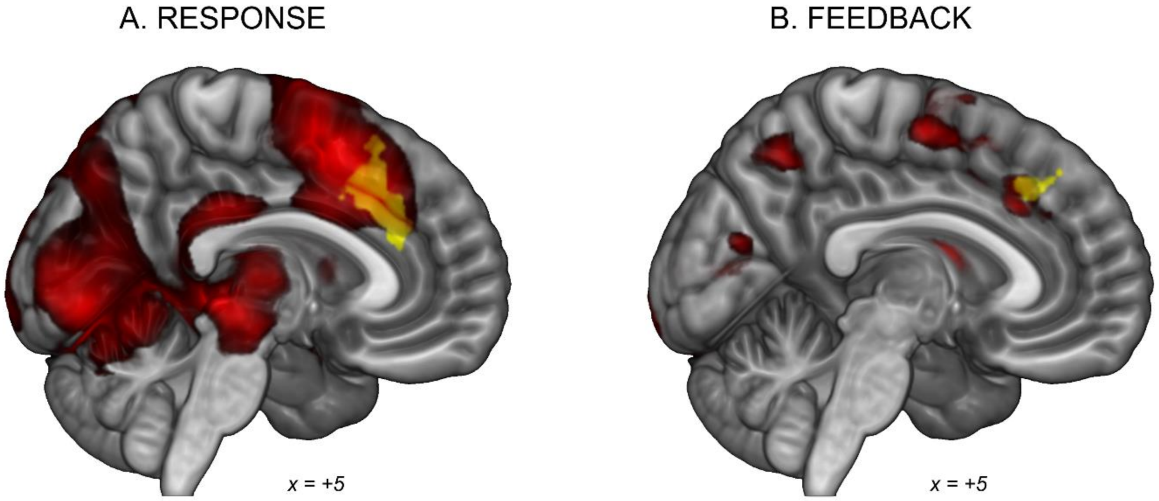

***Supplementary Methods***

To test whether the increase of the amplitude of ERN and FN across learning is the result of neurophysiological processes, or rather the effect of a decreasing number of errors, we have carried out additional data analysis. To balance the number of errors at each learning stage, we randomly selected 4 response-related and 4 feedback-related EEG epochs for each learning time-point. Further, we performed statistical analysis, using the same factors as described in the Methods section (i.e. the same ERP ratings, the same ANOVA model, etc.) on this randomly reduced data.

The obtained results clearly show, that even when we balance the number of errors across the learning time-points, the relationship we describe in our manuscript stands. For the ERN (Fig. 1) there is a statistically significant increase in the amplitude as learning progresses ($F_{(3, 159)}=5.91$, $p<0.001$), and the same holds true for the FN ($F_{(3, 159)}= 8.19$, $p<0.001$) (Fig. 2).

Taken together, this analysis confirms that the effects we report in our manuscript are valid and not affected by the decreasing number of erroneous trials.

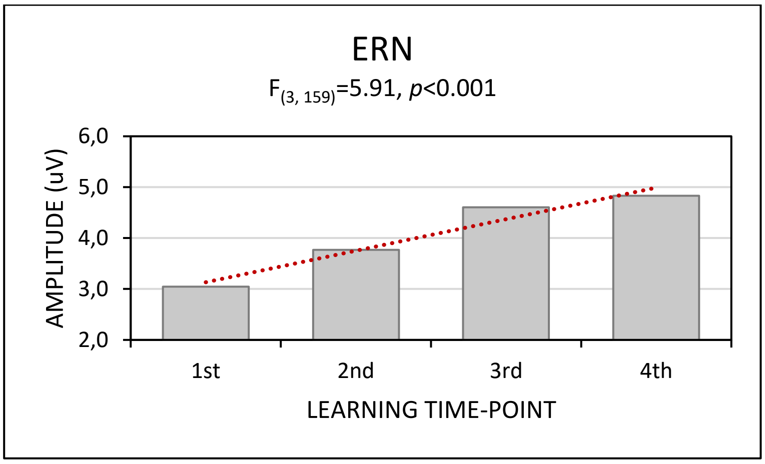


*Figure 1 The absolute amplitude of ERN for 4 learning time-points extracted from the randomly reduced datasets.*

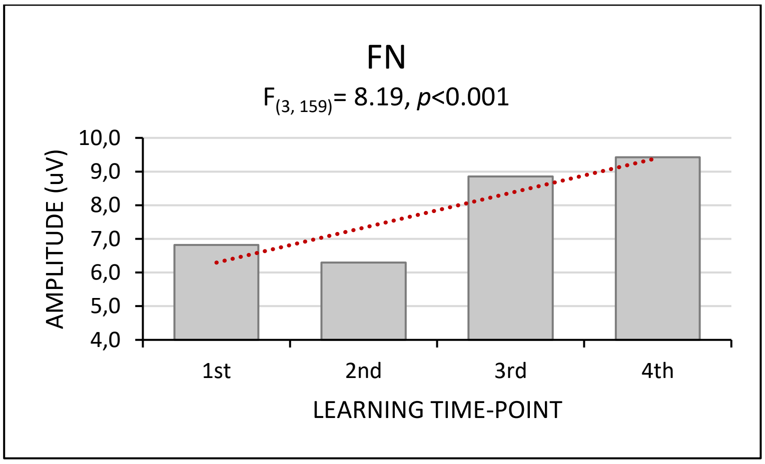


*Figure 2 The absolute amplitude of FN for 4 learning time-points extracted from the randomly reduced datasets*
